# Brown tumor diagnosed three years after parathyroidectomy in a patient with nail-patella syndrome: A case report

**DOI:** 10.1016/j.bonr.2018.100187

**Published:** 2018-12-10

**Authors:** Naoya Toriu, Toshiharu Ueno, Hiroki Mizuno, Akinari Sekine, Noriko Hayami, Rikako Hiramatsu, Keiichi Sumida, Masayuki Yamanouchi, Eiko Hasegawa, Tatsuya Suwabe, Junichi Hoshino, Naoki Sawa, Kenmei Takaichi, Takeshi Fujii, Tomoka Hasegawa, Norio Amizuka, Motoko Yanagita, Yoshifumi Ubara

**Affiliations:** a Nephrology Center, Department of Rheumatology, Toranomon Hospital, Tokyo, Japan; b Department of Pathology, Toranomon Hospital, Tokyo, Japan; c Okinaka Memorial Institute for Medical Research, Tokyo, Japan; d Hard Tissue Developmental Biology Department Graduate School of Dental Medicine, Faculty of Dental Medicine, Hokkaido University, Sapporo, Japan; e Department of Nephrology, Kyoto University Graduate School of Medicine, Kyoto, Japan

**Keywords:** PTX, parathyroidectomy, HPT, hyperparathyroidism, HD, hemodialysis, BAP, bone alkaline phosphatase, BMD, bone mineral density, SD, standard deviation, TRAP, tartrate-resistant acid phosphatase, RANKL, receptor for activator of nuclear factor-κB ligand, γ-GTP, γ-glutamyl transpeptidase, PTH, parathyroid hormone, STIR, short-tI inversion recovery, Brown tumor, Parathyroidectomy, Hyperparathyroidism, Tartrate-resistant acid phosphatase, Cathepsin K, Nail-patella syndrome

## Abstract

We report a 48-year-old Japanese man with a brown tumor of the right distal tibia. At the age of 25 years, hemodialysis was initiated due to nail-patella syndrome. Severe secondary hyperparathyroidism and osteoporosis progressed over time, so parathyroidectomy was performed at age 45. Spontaneous fracture of the right distal tibia occurred suddenly at age 48. Imaging studies revealed a bone tumor-like lesion and surgery was performed. The resected specimen was a brown mass consisting of multinucleated giant cells on a fibrous tissue background, and these findings were consistent with a diagnosis of brown tumor. Immunohistochemistry revealed that multinucleated giant cells near areas of bone matrix were positive for tartrate-resistant acid phosphatase and cathepsin K, but the majority of the giant cells in the lesion were negative for these markers. Even after parathyroidectomy, brown tumor should be considered in the differential diagnosis of bone tumor-like lesions in patients on long-term dialysis. This case suggests that osteoclast activation may not contribute to development of brown tumors, although these lesions are generally considered to arise from subperiosteal bone resorption related to osteoclast overactivity in patients with hyperparathyroidism.

## 1 Introduction

Brown tumor has long been considered to be a bone lesion that arises in the setting of osteoclast overactivity, such as in patients with hyperparathyroidism, and this lesion is also known as osteitis fibrosa cystica (Alfawareh et al., 2015). Histologically, brown tumors feature giant cell proliferation against a background of hemosiderin-rich fibrous tissue. Excessive proliferation of osteoclasts to produce giant cells has been suggested to contribute to formation of brown tumors (Garcia and KMaSA, 1999). These tumors are usually painless and are found incidentally, but may cause damage to adjacent structures or compressive symptoms such as pain, peripheral neuropathy, and myelopathy (Hamouda et al., 2012). Moreover, brown tumors increase the risk of fracture if they develop in weight-bearing bones (Wallace et al., 2013).

In non-urgent cases, symptomatic brown tumors can be treated by medical or surgical control of hyperparathyroidism (Pechalova and Poriazova, 2013). When a brown tumor is resistant to conservative treatment, parathyroidectomy (PTX) can arrest tumor progression and lead to a decrease in size (Hamouda et al., 2012). On the other hand, regression of a brown tumor after PTX is sometimes slow, and surgical resection of brown tumor may need to be performed (Leal et al., 2006). However, there have been no reports of symptomatic brown tumors diagnosing after PTX. Also, whether the giant cells in brown tumors possess the functions of osteoclasts has not been evaluated.

Here we report a patient on long-term hemodialysis for nail-patella syndrome, in whom a symptomatic brown tumor was diagnosed three years after PTX for secondary hyperparathyroidism. Immunohistochemical evaluation of this patient's brown tumor was performed.

## 2 Case report

**Fig. 1:**
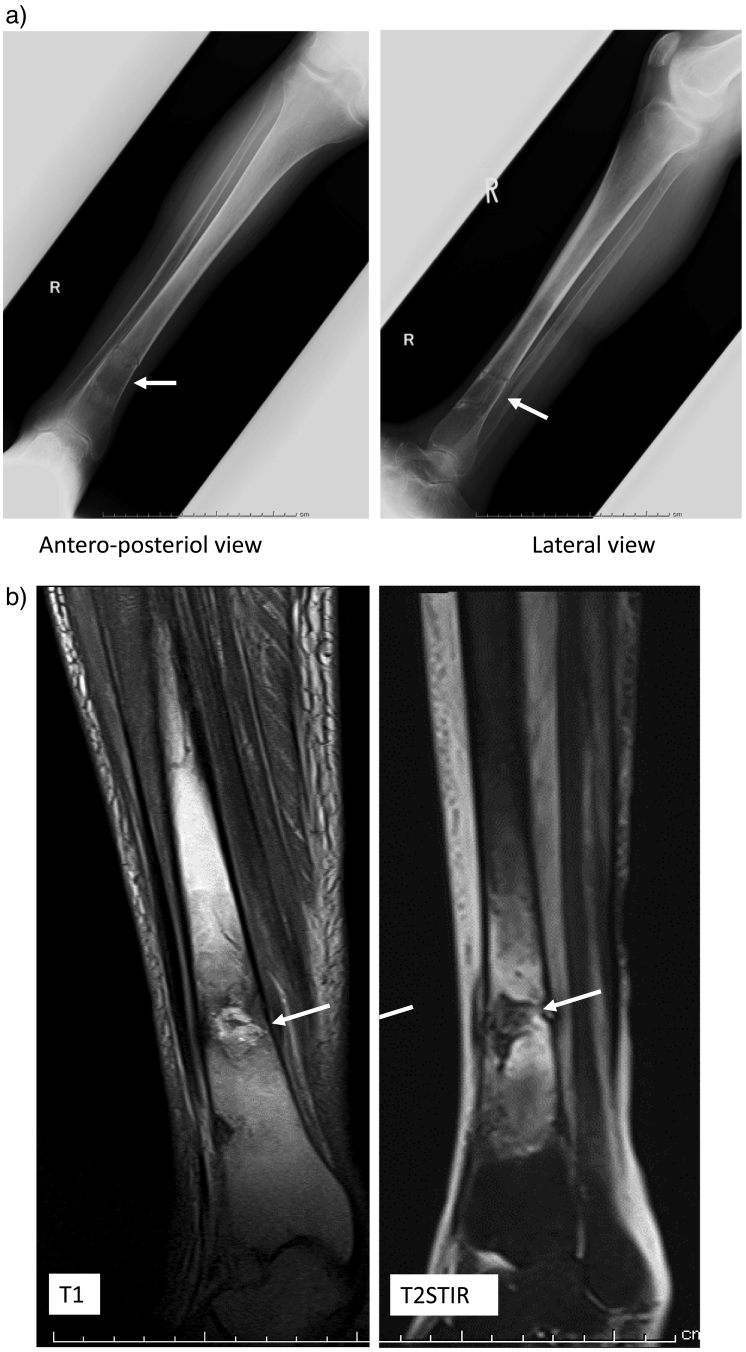
a: Plain radiograph shows a radiolucent lesion associated with a fracture line in the distal right tibia. b: Magnetic resonance imaging reveals a bone tumor-like lesion and fracture line in the right tibia. MRI T1-weighted images shows hyper intensity, T2-weighted images shows hypo intensity, and T2STIR(short-tI inversion recovery) shows hypo intensity, but was inconsistent with malignant bone tumor pattern that T1 shows low intensity to iso intensity, and T2 shows high intensity to iso intensiy.

A 48-year-old Japanese man was admitted to our hospital for evaluation of pain in the distal right tibia. Nephrotic syndrome occurred at the age of 4 years. Subsequently, nail-patella syndrome was diagnosed due to the presence of nail dysplasia, patellar aplasia, and bilateral iliac horns on a pelvic radiograph. Hemodialysis (HD) was initiated at the age of 25 years. A liver tumor was detected by ultrasonography at the age of 43 years and hepatectomy was done. Histological examination showed a moderately differentiated hepatocellular carcinoma with normal background liver architecture. Markers of hepatitis B virus and hepatitis C virus were negative. At the age of 45 years, PTX was performed because hyperparathyroidism became resistant to medical management, including maxacalcitol and cinacalcet, and osteoporosis progressed (Table 1). Before PTX, intact parathyroid hormone (iPTH) was 568 pg/mL,bone alkaline phosphatase (BAP) was 43 μg/L, and tartrate-resistant acid phosphatase (TRAP)-5b was 1370 mU/dL. A total of 4 parathyroid glands were removed, and part of the smallest gland was re-implanted in the right forearm muscle. iPTH decreased to 24 pg/ml on postoperative day 1, but returned to 229 pg/ml after 1 year. At the age of 48 years, pain and swelling suddenly developed at the distal right tibia with no precipitating cause when the patient stood up. A plain radiograph showed a radiolucent lesion with a fracture line in the distal right tibia (Fig. 1a). Magnetic resonance imaging revealed a bone tumor-like mass lesion associated with a fracture line (Fig.1b).

**Table 1:** Laboratory findings.

Age	45	48	Normal range
Year	2012	2015	
White blood cell	8000	4500	3200–7900/μL
Hemoglobin	9.2	8.8	11.3–15.0g/dL
Hematocrit	30	27.5	34.0–46.3%
Platelate	239	178	155–350 ∗ 10^3^/μL
Total protein	6.6	6.3	6.9–8.4 g/dL
Albumin	2.4	2.3	3.9–5.2 g/dL
Aspartic aminotransferase	20	15.0	13–33 IU/L
Alanine transaminase	21	15	8–42 IU/L
Lactate dehydrogenase	251	323	119–229 IU/L
Alkaline phosphatase	624	418	117–350 IU/L
Bone alkaline phosphatase	43	25.2	3.7–20.9 μg/L
gamma-Glutamyl transferase	242	179	9–109 IU/L
TRAP-5b	n/a	818	170–590 mU/dL
Urea nitrogen	61	53.0	8–21 mg/dL
Creatinine	9.7	8.2	0.46–0.78 mg/dL
Uric acid	8.4	7.5	2.5–7.0 mg/dL
Na	141	143	139–146 mmol/L
K	5.3	5.2	3.7–4.8 mmol/L
Cl	101	101	101–109 mmol/L
Corrected calcium	9.5	10.0	8.7–10.1 mg/dL
P	4.9	4	2.8–4.6 mg/dL
Fe	27	29	80–120 μg/dL
UIBC	271	195	173–263 μg/dL
Ferritin	11	50	10–190 μg/L
β2 microglobulin	41.7	24.7	0.5–2.0 mg/mL
CRP	2.8	1.6	0.0–0.3 mg/dL
Intact PTH	568	162	15–65 pg/ml

Bone mineral density (Z score)			
Lumbar (SD)	−0.6	ND	g/cm^3^ (SD)
Distal radius (SD)	−6.4	ND	g/cm^3^ (SD)
Proximal femoral (SD)	−3.6	ND	g/cm^3^ (SD)

On admission, his blood pressure was 156/106 mm Hg. Laboratory tests gave the following results (Table 1): BAP, 7.0 μg/L, TRAP-5b, 86 mU/dL; corrected Ca, 10.0 mg/dL; P, 4.0 mg/dL; and iPTH, 162 pg/mL. The lumbar bone mineral density (BMD) measured by dual X-ray absorptiometry was 0.671 g/cm^3^ (Z score: −0.6 SD), the distal radial BMD was 0.400 g/cm^3^ (Z score: −6.4 SD), and the proximal femoral BMD was 0.453 g/cm^3^ (Z score: −3.6 SD). He was taking calcium carbonate (3 g/day) and lanthanum carbonate hydrate (1500mg/day). Genetic testing revealed mutation of the LMX1β gene, which is the typical cause of nail-patella syndrome. The surgical open resection and internal fixation of the distal right tibia brown tumor was performed under general anesthesia.

## 3 Histopathology of the surgical specimen

**Fig. 2:**
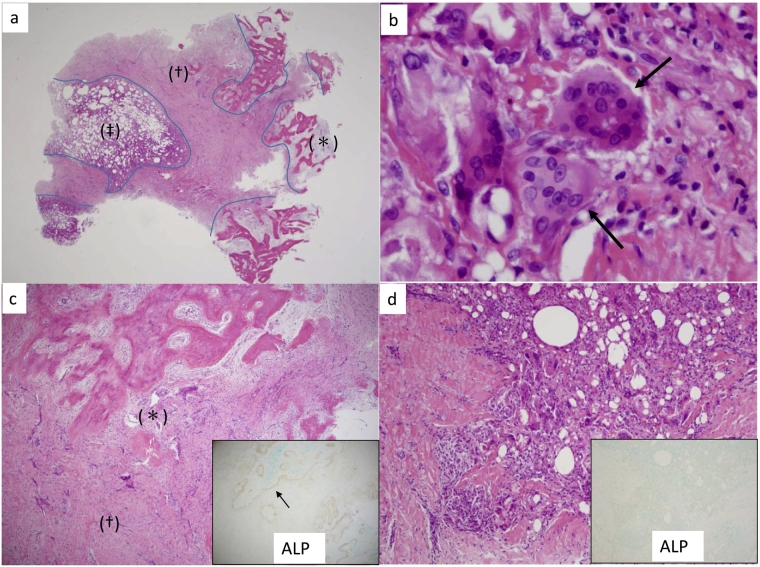
Histology of the brown tumor. a: Hematoxylin eosin (HE) staining reveals that bone matrix (*) has been replaced by brown-colored tissue (the brown tumor) (†) and marrow fat (‡). b: HE staining shows a multinucleated giant cell (arrow) against a background of fibrous tissue composed of spindle-shaped fibroblasts. c: HE staining reveals a brown tumor (†) near the bone matrix (*). Inset: Mononuclear cells located close to the bone matrix are positive for alkaline phosphatase (ALP) by immunohistochemistry (arrow), indicating the presence of osteoblast-like cells. d: HE staining of the brown tumor distant from the bone matrix. Inset: Immunohistochemistry does not identify any mononuclear cells positive for ALP, indicating the absence of osteoblast-like cells.

**Figure 3:**
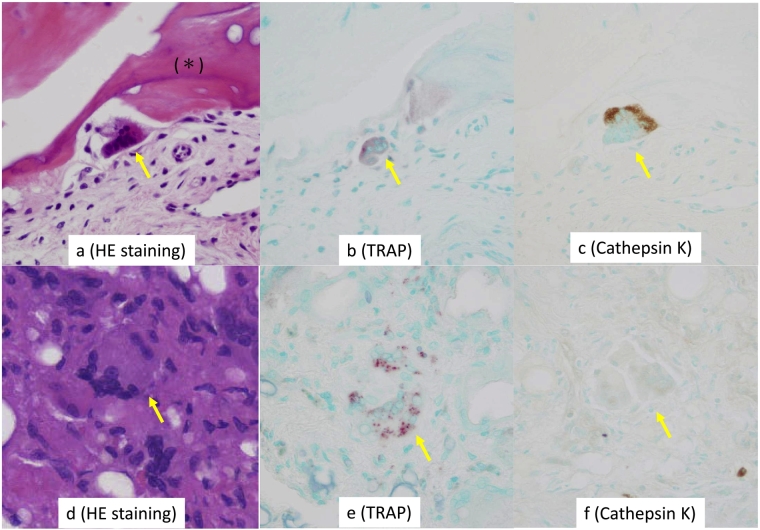
Immunohistochemistry of giant cells in the brown tumor. a: Giant cell (arrow) near the bone matrix (*). b: Giant cells (arrow) near the bone matrix is positive for tartrate-resistant acid phosphatase (TRAP). c: Giant cell near the bone matrix are positive for cathepsin K (arrow). d: Giant cells (arrow) distant from the bone matrix. e: Giant cell (arrow) distant from the bone matrix are negative for TRAP. f: Giant cells (arrow) distant from the bone matrix is negative for cathepsin K.

On light microscopy, brown tumor was diagnosed because the tumor-like lesion revealed characteristic brown-colored tissue by hematoxylin eosin stain and marrow fat. In the brown tumor, mononuclear cells and multinucleated giant cells were seen against a background of fibrous tissue formed by spindle-shaped fibroblasts (Fig. 2). Immunohistochemistry showed that mononuclear cells located near areas of bone matrix were positive for alkaline phosphatase (ALP), indicating the presence of osteoblast-like cells with osteoblastic activity. In contrast, mononuclear cells distant from the bone matrix were negative for ALP, indicating these were not osteoblast-like cells (Fig. 2). A few multinucleated giant cells near the bone matrix showed positivity for tartrate-resistant acid phosphatase (TRAP) and cathepsin K, indicating the presence of osteoclast-like cells with osteoclastic activity. Inf contrast, the majority of the multinucleated giant cells in the tumor (distant from the bone matrix) were negative for TRAP and cathepsin K, indicating that these cells did not possess osteoclastic activity (Fig. 3).

## 4 Clinical course

**Fig. 4:**
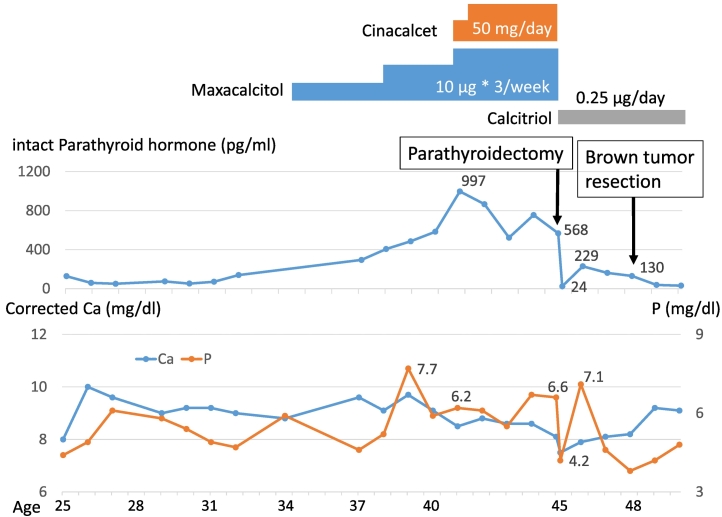
Clinical course: At the age of 45, parathyroidectomy was performed. At the age of 48, spontaneous fracture of the right distal tibia due to brown tumor occurred and brown tumor resection was performed.

The patient did well after surgery and he was free of symptoms at 2 years postoperatively (Fig. 4). However, he died of cerebral hemorrhage at the age 50.

## 5 Discussion

The frequency of brown tumors is slightly higher in patients with primary than secondary hyperparathyroidism (3% vs. 2%), but the number of patients with secondary HPT is larger, so brown tumors are mainly associated with secondary HPT (Chew and Huang-Hellinger, 1993). To the best of our knowledge, there have been no previous reports of a symptomatic brown tumor causing bone fracture after PTX.

Nail-patella syndrome is an autosomal dominant disorder due to mutations of the LMX1B gene. The pathognomonic symptoms are small or absent patellae and dysplastic or absent fingernails and toenails (Witzgall, 2017). 5 – 10% of these patients develop nephrotic range proteinuria in childhood and show rapid progression to end-stage renal disease (Lemley, 2009). A relation between brown tumor and nail-patella syndrome has not been reported.

Desai et al. studied the ultrastructure of two brown tumors in patients with hyperparathyroidism. They reported that these tumors contain multinucleated giant cells and fibroblasts. The giant cells have ultrastructural features similar to osteoclasts, including numerous mitochondria, dilated rough endoplasmic reticulum, and short filopodia, but the typical ruffled borders of osteoclasts are not seen. Accordingly, Desai et al. suggested that the giant cells in brown tumors may be inactive osteoclasts (Desai and Steiner, 1990). Okada et al. reported on the histology of brown tumor, stating that most of the giant cells were strongly positive for CD68 and weakly positive for vimentin and lysozyme (Okada et al., 2000). TRAP has been reported to show a close relation to bone-resorbing osteoclasts and activated macrophages because of its phosphatase activity and generation of reactive oxygen species, but TRAP has not previously been evaluated in brown tumors (Halleen et al., 2003). Hiramatsu et al. reported that TRAP and cathepsin K are markers of osteoclast-like cells (Hiramatsu et al., 2013). They stated that multinucleated giant cells showed definite positivity for TRAP and cathepsin K, and were considered to have osteoclastic activity, unlike our case.

The brown tumor shows radiolucency on x-ray, and shows an existence of multinucleated giant cells histologically, and has been considered a bone lesion that has a close relation with excess osteoclast activity due to hyperparathyroidism, but whether multinucleated giant cell shows osteoclast marker on symptomatic brown tumor has not yet been demonstrated. Temporal relationship between development of Brown tumor and its progression until bone fracture occurs will become the future problem. Also, from radiological standpoint, it may become one of the differential diagnosis of bone tumor in a middle-aged male.

On this case, MRI T1-weighted images shows hyper intensity, T2-weighted images shows hypo intensity, and T2STIR(short-tI inversion recovery) shows hypo intensity, and was inconsistent with malignant bone tumor pattern that T1 shows low intensity (to iso intensity), and T2 shows high intensity (to iso intensiy).

In conclusion, we evaluated a patient with a brown tumor that became symptomatic at 3 years after PTX. Histological examination of the tumor showed multinucleated giant cells on a background of fibrous tissue. Immunohistochemistry demonstrated that only multinucleated giant cells near the bone matrix were positive for TRAP and cathepsin K, while the majority of the giant cells in the tumor were negative for osteoclastic activity. These tumors have been considered to represent subperiosteal bone resorption related to excessive osteoclast activity in the setting of hyperparathyroidism. However, our report suggests that osteoclast activation may not be the sole pathologic mechanism known for development of Brown tumor, although this needs more evidence to be suggested.

## Statement of ethics

The present report was produced in conformity with the Declaration of Helsinki, and the patient gave consent for this case report to be published.

## Disclosures

The authors declare no competing financial interests.

The authors also declare that they have no conflicts of interest.

## References

[bb0005] Alfawareh M.D., Halawani M.M., Attia W.I., Almusrea K.N. (2015). Brown tumor of the cervical spines: a case report with literature review. Asian Spine J..

[bb0010] Chew F.S., Huang-Hellinger F. (1993). Brown tumor. AJR Am. J. Roentgenol..

[bb0015] Desai P., Steiner G.C. (1990). Ultrastructure of brown tumor of hyperparathyroidism. Ultrastruct. Pathol..

[bb0020] Garcia R.A., KMaSA (1999). Primary hyperparathyroidism. Robbins Pathology.

[bb0025] Halleen J.M., Raisanen S.R., Alatalo S.L., Vaananen H.K. (2003). Potential function for the ROS-generating activity of TRACP. J. Bone Miner. Res..

[bb0030] Hamouda M., Handous I., Ben Dhia N. (2012). Brown tumors in dialyzed patients with secondary hyperparathyroidism: report of 16 cases. Hemodialysis International Symposium on Home Hemodialysis.

[bb0035] Hiramatsu R., Ubara Y., Hayami N. (2013). Occurrence of new bone-like tissue formation in uremic tumoral calcinosis. Bone.

[bb0040] Leal C.T., Lacativa P.G., Gomes E.M. (2006). Surgical approach and clinical outcome of a deforming brown tumor at the maxilla in a patient with secondary hyperparathyroidism due to chronic renal failure. Arq. Bras. Endocrinol. Metabol..

[bb0045] Lemley K.V. (2009). Kidney disease in nail-patella syndrome. Pediatr. Nephrol..

[bb0050] Okada H., Davies J.E., Yamamoto H. (2000). Brown tumor of the maxilla in a patient with secondary hyperparathyroidism: a case study involving immunohistochemistry and electron microscopy. J. Oral Maxillofac. Surg..

[bb0055] Pechalova P.F., Poriazova E.G. (2013). Brown tumor at the jaw in patients with secondary hyperparathyroidism due to chronic renal failure. Acta Med. (Hradec Kralove).

[bb0060] Wallace E., Day M., Fadare O., Schaefer H. (2013). Pathologic femur fracture due to a brown tumor in a patient with secondary hyperparathyroidism and vitamin D-resistant rickets. Am. J. Kidney Dis..

[bb0065] Witzgall R. (2017). Nail-patella syndrome. Pflugers Arch. - Eur. J. Physiol..

